# Analysis of Brain Age Gap across Subject Cohorts and Prediction Model Architectures

**DOI:** 10.3390/biomedicines12092139

**Published:** 2024-09-20

**Authors:** Lara Dular, Žiga Špiclin, for the Alzheimer’s Disease Neuroimaging Initiative, the Australian Imaging Biomarkers and Lifestyle Flagship Study of Ageing

**Affiliations:** University of Ljubljana, Faculty of Electrical Engineering, Tržaška cesta 25, 1000 Ljubljana, Slovenia

**Keywords:** brain age, brain age gap, deep regression models, multiple sclerosis, Alzheimer’s dementia, Parkinson’s disease, sleep apnea, diabetes, UK Biobank

## Abstract

**Background:** Brain age prediction from brain MRI scans and the resulting brain age gap (BAG)—the difference between predicted brain age and chronological age—is a general biomarker for a variety of neurological, psychiatric, and other diseases or disorders. **Methods:** This study examined the differences in BAG values derived from T1-weighted scans using five state-of-the-art deep learning model architectures previously used in the brain age literature: 2D/3D VGG, RelationNet, ResNet, and SFCN. The models were evaluated on healthy controls and cohorts with sleep apnea, diabetes, multiple sclerosis, Parkinson’s disease, mild cognitive impairment, and Alzheimer’s disease, employing rigorous statistical analysis, including repeated model training and linear mixed-effects models. **Results:** All five models consistently identified a statistically significant positive BAG for diabetes (ranging from 0.79 years with RelationNet to 2.13 years with SFCN), multiple sclerosis (2.67 years with 3D VGG to 4.24 years with 2D VGG), mild cognitive impairment (2.13 years with 2D VGG to 2.59 years with 3D VGG), and Alzheimer’s dementia (5.54 years with ResNet to 6.48 years with SFCN). For Parkinson’s disease, a statistically significant BAG increase was observed in all models except ResNet (1.30 years with 2D VGG to 2.59 years with 3D VGG). For sleep apnea, a statistically significant BAG increase was only detected with the SFCN model (1.59 years). Additionally, we observed a trend of decreasing BAG with increasing chronological age, which was more pronounced in diseased cohorts, particularly those with the largest BAG, such as multiple sclerosis (−0.34 to −0.2), mild cognitive impairment (−0.37 to −0.26), and Alzheimer’s dementia (−0.66 to −0.47), compared to healthy controls (−0.18 to −0.1). **Conclusions:** Consistent with previous research, Alzheimer’s dementia and multiple sclerosis exhibited the largest BAG across all models, with SFCN predicting the highest BAG overall. The negative BAG trend suggests a complex interplay of survival bias, disease progression, adaptation, and therapy that influences brain age prediction across the age spectrum.

## 1. Introduction

Brain age, a biomarker representing the estimated age of the brain using machine learning, has been a focal point of neuroscience research for over a decade [1]. Brain age prediction involves training machine learning models to estimate an individual’s age from brain images, typically from raw or quantified T1-weighted magnetic resonance (T1w MR) images. The brain age gap (BAG), defined as the difference between the predicted brain age and the true chronological age, has been shown to differentiate between cohorts of healthy subjects and patients with various neurological, psychiatric, and other diseases or disorders, suggesting its potential utility as a biomarker for disease detection and progress monitoring [2,3].

Neurological diseases have been the primary focus of brain age research, with notable findings across various conditions. For instance, patients with Parkinson’s disease (PD) exhibit an increased brain age compared to their healthy peers [4,5]. Specifically, patients without cognitive impairment have a brain age gap (BAG) of approximately 2 years, whereas those with cognitive impairment exhibit a BAG of over 7 years [6]. Furthermore, studies have shown that patients with mild cognitive impairment (MCI) exhibit an increased brain age of up to 6 years [7]. For those with Alzheimer’s disease (AD), BAG can extend by up to 8 years [8,9] and up to 11 years for patients with frontotemporal dementia [8]. Recent studies also have shown an increased BAG for multiple sclerosis (MS) patients, revealing a substantial BAG across all subtypes [10,11]. Notably, the increase in BAG is particularly pronounced for those with secondary progressive MS (SPMS) [12].

Common systemic diseases and disorders, lifestyle choices, and physical activity also impact brain age and BAG. For instance, increased brain age has been reported for individuals with chronic metabolic diseases, particularly type 2 diabetes mellitus [13,14,15,16,17], which is associated with brain atrophy [18] and white matter hyperintensities [19]. A recent study revealed significant positive associations between brain age and two indices quantifying the obstructive sleep apnea, namely the apnea–hypopnea index and the oxygen desaturation index [20]. For healthy individuals, BAG has been shown to be influenced by lifestyle choices, with smoking and alcohol consumption being associated with increased brain age [16,21]. Conversely, engaging in physical activity [21] and maintaining physical strength [15] are associated with decreased brain age.

With the use of deep learning approaches, the accuracy of brain age prediction models has significantly improved. However, different model architectures have shown varying performance in terms of accuracy, robustness, reproducibility, and (longitudinal) consistency [22]. The choice of the model architecture and its (hyper)parameters, as well as the training/validation procedures may critically affect the predicted BAG between the healthy population and diseased cohorts. Interestingly, Bashyam et al. [3] found that models with a looser fit, characterized by a higher mean absolute error (MAE), provided better separation according to BAG between the diseased subjects and healthy controls than the tighter-fitting, more accurate models (i.e., lower MAE). This suggests that while a model may excel in accurately predicting brain age in healthy individuals, its ability to detect disease-related BAG may be compromised. Therefore, a systematic comparison and statistical evaluation of various models across different subject cohorts, with different diseases and disorders, is essential to accurately estimate BAG and understand how different model architectures, (hyper)parameter settings, and training/validation procedures influence these predictions across diverse subject conditions.

Previous studies on model comparison have primarily focused on brain age prediction for healthy individuals, aiming to achieve the most accurate predictions [22,23,24]. Alternatively, related work has compared BAG across different diseases, but often using a single model architecture [25,26]. While brain age analysis on diseased datasets is well researched, the results are difficult to compare due to variations in training and testing datasets. This study addresses this gap by investigating the differences in estimated BAG across five deep learning models applied to cohorts with various diseases and disorders, including sleep apnea, diabetes, multiple sclerosis, Parkinson’s disease, mild cognitive impairment, and Alzheimer’s disease. We employed rigorous statistical evaluation based on repeated model training and a standardized evaluation protocol. To the best of our knowledge, this is the first study to systematically compare BAG across multiple deep learning models for a diverse range of diseases and disorders.

The contributions of this work are as follows: (i) a comprehensive analysis of the brain age gap (BAG) across five diseases or disorders—sleep apnea, diabetes, multiple sclerosis, Parkinson’s disease, mild cognitive impairment, and Alzheimer’s disease—as well as in a healthy population; (ii) a comparison of the performance of five state-of-the-art deep learning model architectures: 2D/3D VGG, RelationNet, ResNet, and SFCN; (iii) a rigorous evaluation protocol that incorporates repeated training of the brain age prediction models to ensure robustness; and (iv) a statistical evaluation framework based on linear mixed-effects models to rigorously assess the results across different models and disease conditions.

This paper is organized as follows: Section 2 presents the MRI datasets, deep learning models, and the statistical methods used for results analysis. Section 3 details the experiments and their results. Section 4 provides a discussion of the findings, and finally, Section 5 concludes with the overall discussion and implications of the study.

## 2. Materials and Methods

### 2.1. Datasets

#### 2.1.1. Multi-Site Train Dataset

For training the models, we have gathered seven public datasets, comprising a multi-site dataset of 2504 healthy subjects (Table 1). All models were trained on 2012 subjects and tested on a dataset of 247 subjects. Additionally, a validation dataset of 245 subjects was used for hyperparameter tuning.

#### 2.1.2. Diseased Datasets

For the assessment of the brain age gap in the diseased subject cohorts, we included two datasets that were not used during model training. The first is a subset of the UK Biobank dataset [31] and includes healthy controls (HC) and four groups with various diseases or disorders, namely sleep apnea (SA), diabetes (Dia), multiple sclerosis (MS), and Parkinson’s disease (PD). The subgroups were formed based on self-reported disease and health ratings. To exclude co-morbidity, subjects with schizophrenia, neurological injury or trauma, psychological or psychiatric problems, brain or intracranial abscess, chronic or degenerative neurological problems, dementia or cognitive impairment, epilepsy, head or spinal injury, and depression were excluded, except for the subgroups of MS and PD, where patients who reported depression were included, due to the well-known link between depression and the two chronic diseases [32,33]. Healthy controls were selected as subjects who reported good or excellent self-reported health and none of the aforementioned diseases, and were used for model calibration. Additionally, the self-reported health status was taken into account in the diabetes subgroup, where subjects with fair, good, or excellent health were selected. The aforementioned five subsets are disjoint, i.e., each subject belongs to one and only one subset.

To study the effect of brain age on mild cognitive impairment (MCI) and Alzheimer’s dementia (AD), we included the AIBL dataset. Data were collected by the AIBL study group, whereas the AIBL study methodology has been reported previously [34]. The dataset included multiple scans for each subject, of which the last MRI was included in the dataset. The details of diseased datasets are presented in Table 2.

#### 2.1.3. T1-Weighted Image Preprocessing

The T1-weighted MR image preprocessing pipeline was selected as the best of the four tested pipelines in the context of brain age estimation in our previous publication [35]. The raw T1w image was subjected to adaptive non-local means denoising (Adaptive non-local means denoising Version 2.0: https://github.com/djkwon/naonlm3d, accessed on 13 July 2024) [36]. The denoised image was then registered to the Montreal Neurological Institute (MNI) atlas space using an affine transformation via NiftyReg (NiftyReg Software, Centre for Medical Image Computing at University College London, London, UK: http://cmictig.cs.ucl.ac.uk/wiki/index.php/NiftyReg, accessed on 13 July 2024) [37]. Prior to registration, we applied an intensity inhomogeneity correction using the N4 algorithm (N4 algorithm, ANTS software, University of Pennsylvania Richards Medical Research Laboratories, Philadelphia, USA: https://manpages.debian.org/testing/ants/N4BiasFieldCorrection.1.en.html, accessed on 13 July 2024) [38] to enhance the accuracy of the registration. The intensity-corrected MR image was used only for computing the transformation and then discarded.

Further, the denoised image was resampled into MNI space using the computed transformation matrix and sinc interpolation to achieve a uniform size of 193×229×193 with isotropic 1 mm spacing. Next, a two-step adjustment of the grayscale values, including intensity windowing and intensity inhomogeneity correction using the N4 algorithm, was performed. Finally, the empty space surrounding the head was cropped to focus on relevant brain structures, reducing the processed T1w images to a size of 157×189×170. The details and implementation of the preprocessing are specified in [35].

### 2.2. Model Architectures

**Figure 1:**
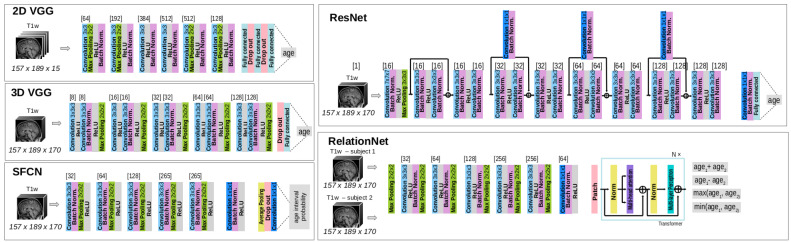
Model architectures of five implemented brain age prediction models.

Five deep learning model architectures, previously used in the related brain age literature, were (re)implemented as follows: (1) 2D VGG-based model proposed by Huang et al. [39] (**2D VGG**), (2) 3D VGG-based model proposed by Cole et al. [40] (**3D VGG**) (2D/3D VGG: implementation available at https://github.com/AralRalud/BASE, accessed on 13 July 2024), (3) Deep Relation Learning model utilized by He et al. [41] (**RelationNet**) (RelationNet: implementation available at https://github.com/shengfly/BrainAgingNet/blob/main/Relation-Transformer.pyg, accessed on 13 July 2024), (4) Residual Network utilized by Hu et al. [42] and Dartora et al. [43] (**ResNet**) (ResNet: implemented in MONAI version 0.8 or later.) (Dartora used ResNet-26, while we used a shallower network, ResNet-18.), and (5) Simple Fully Convolutional Network utilized by Peng et al. [44] (**SFCN**) (SFCN: implementation available at https://github.com/ha-ha-ha-han/UKBiobank_deep_pretrain, accessed on 13 July 2024). Model architectures are depicted in Figure 1.

While the output of the 2D VGG, 3D VGG, and ResNet models is a single scalar value, obtained using a fully connected layer with a linear activation function representing the predicted age of an individual, RelationNet uses an MLP with a linear activation to produce four scalar values: the sum, difference, minimum, and maximum of the ages for two input images from two subjects. The individual ages are then derived by solving a system of equations using the sum and difference values [41]. In contrast, the SFCN model uses Conv 1×1×1 layers with softmax activation to output a vector of class probabilities, with each class representing a two-year interval, such as [18, 20], (20, 22], (22, 24], and so on. To obtain a single scalar age prediction, we calculate the weighted average of the midpoint of each interval, using these class probabilities as the weights [44].

All models were implemented and trained/validated in PyTorch 1.4.0 and Python 3.6.8. For the ResNet model, we used the implementation provided in the MONAI 0.8 library.

### 2.3. Model Setup and Evaluation Protocols

#### 2.3.1. Model Training

Each model was subject to hyperparameter tuning, selecting the optimal set of values based on the best accuracy (i.e., MAE) achieved on the multi-site validation dataset. Training protocols were specific to each model architecture, in the sense that they were implemented as close to the original version as discernible from the associated research paper and/or code repository. The exact values of the hyperparameter for each model are reported in Table 3.

The choice of the loss function was based on the specific task formulation, whether it was a regression or classification problem. The SFCN model was trained using Kullback–Leibler divergence (KLD), while the other models utilized the L1 loss function. For optimization, we employed the Stochastic Gradient Descent (SGD) algorithm.

The input images were subjected to data augmentation using the following operators and probabilities: scaling with padding/cropping to maintain the same input image size (p=0.3), translational shift (p=0.3), and left/right axis flip (p=0.5).

#### 2.3.2. Evaluation

Each model architecture was trained on the same data using five instances of randomly generated weights, yielding five sets of model weights for each architecture. The five brain age predictions per subject obtained for a particular model architecture were then combined into a mean ensemble, yielding one prediction per model architecture.

For the evaluation of model performance, we adhered to the Brain Age Standardized Evaluation (BASE) guidelines [22] for cross-sectional datasets. We assessed model performance using two accuracy metrics, namely mean absolute error (MAE) and mean error (ME), defined as:

$$MAE=\frac{1}{N}\sum_{i=1}^{N}|y_{i}^{\prime }-y_{i}|$$

and

$$ME=\frac{1}{N}\sum_{i=1}^{N}\left(y_{i}^{\prime }-y_{i}\right),$$

where $y_{i}$ denotes the true age, $y_{i}^{\prime }$ the predicted age of the *i*-th subject, and *N* is the sample size.

Additionally, we computed the robustness metric maximal MAE (mMAE) defined as

$$mMAE=\max_{j}\frac{1}{N_{j}}\sum_{i=1}^{N_{j}}1_{\left\{y_{i}\in \left[c_{j},c_{j+1}\right)\right\}}|y_{i}^{\prime }-y_{i}|,$$

where $N_{j}$ is the number of samples from the interval $\left[c_{j},c_{j+1}\right)$ and $\sum_{j}N_{j}=N$, which was calculated by taking the maximum MAE over age intervals [45,55],(55,65],(75,85], and (85,100]. Note that the age intervals are specifically adapted to the age ranges appearing in the test data as described in Table 2.

#### 2.3.3. Statistical Analysis

For the statistical evaluation, we applied the linear mixed-effects models (LMEMs) to characterize the difference in brain age prediction error between subgroups. The LMEM included model architecture, age, diseased group, and their two- and three-way interactions as fixed effects, and subject ID as a random effect. The significance of fixed effects was confirmed using an analysis of variance (ANOVA) test on the fitted model and pairwise comparisons between the levels of the fixed factor using the estimated marginal means (EMM) method, with a Tukey adjustment for multiple comparisons. The LMEM analysis was conducted in R version 4.0.4, using ‘lme4’ package version 1.1.26, ‘lmerTest’ version 3.1.3, and ‘emmeans’ version 1.5.4.

## 3. Results

An assessment of BAG and its comparison across different deep learning model architectures and subject cohorts was performed. The first subsection (Section 3.1) aims to validate the baseline performance of the models by ensuring an adequate fit on a multi-site dataset and on the HC subset of the UK Biobank dataset, which was not used during model training (Section 3.2).

After establishing the baseline performance on the HCs, the main experiments (Section 3.2) focus on analyzing the differences in BAG between HCs and various diseased subsets, highlighting the impact of different model architectures on these predictions.

### 3.1. Baseline Performance on Dataset of Healthy Cohorts

Models were trained and evaluated on the multi-site dataset, with results for accuracy and robustness metrics presented in Table 4. While all models showed an adequate fit, there were notable differences in performance. Specifically, the 2D VGG performed the worst, displaying the highest MAE and ME, alongside the greatest standard deviation (SD), indicating high variability in predictions. The 3D VGG and ResNet achieved MAE values below 3 years, outperforming RelationNet and SFCN, suggesting better reliability for brain age prediction in the multi-site dataset.

The LMEM was used to analyze prediction error using age, model architecture, and their interaction as fixed effects, with subject ID as a random factor. The results of ANOVA revealed significant main effects and interactions (p<0.0001), while the marginal means analysis showed significant differences in prediction error between the 2D VGG model and both the RelationNet (p=0.0376) and the 3D VGG (p=0.0011) models.

The estimated age trend slope coefficients and their 95% confidence intervals (CIs) for each model are shown in Table 4. All estimated age trend slopes are negative, meaning that all models exhibit regression to the mean, estimating older subjects as younger than their actual age and younger subjects as older than their actual age. The 2D VGG’s trend slope coefficient of −0.1021 suggests a 4-year prediction error difference between 80- and 40-year-old subjects, while the 3D VGG, with a trend slope coefficient closest to 0, yields a difference of approximately 1.5 years.

**Figure 2:**
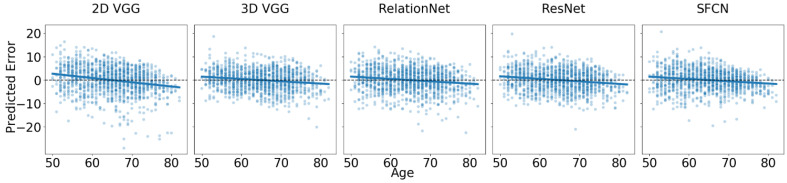
True age and prediction error for all five models on the HC subsample of the UK Biobank database. The blue line represents a fitted linear regression line, illustrating the relationship between age and prediction error for each model.

In the new site dataset, UK Biobank, which was not used during model training, we observed a drop in accuracy of approximately 0.6 years on average before offset correction, which was reduced to about 0.15 years on average after correcting for the offset. The results are reported in Figure 2 and Table 5. It is important to note that the offset correction did not affect the trends presented in Table 5.

Despite a good fit, we observed regression to the mean, which was more pronounced than that seen in the multi-site test dataset. The trend slope coefficients for the main effect of age showed that the 2D VGG model had the slope furthest from zero (i.e., −0.18), while the SFCN (−0.098) and 3D VGG (−0.099) models had slope coefficients closest to zero. For example, the difference in predicted error between 80- and 40-year-old subjects is approximately 4 years for the best-performing models. Pairwise comparisons of the estimated trend slope coefficients revealed statistically significant differences between the 2D VGG model and each of the other models (p<0.001), but not between any of the other models (p>>0.05).

For the healthy controls of the UK Biobank dataset, an LMEM was fitted with predicted error as the outcome, age, model architecture, and their interaction as fixed effects, and subject ID as a random effect. All estimated coefficients were statistically significantly different from zero (p<0.001). The ANOVA indicated that all fixed effects and interactions were statistically significant (p<0.001). Since we individually corrected the offset for each model by subtracting the mean error to achieve a resulting mean error of zero, we did not estimate the marginal means between architectures (as they are all zero).

### 3.2. Brain Age Gap for Diseased Subgroups

To estimate the BAG, we applied all model ensembles to the T1w MR images in the diseased cohorts. The predictions of each model were bias-corrected by adding or subtracting a constant value to ensure that the mean error on the HC subset equaled zero. To statistically evaluate BAG and its relative trend with respect to age (i.e., how the BAG changes as a function of the actual age), we fitted an LMEM with model architecture, age, and cohort (i.e., diseased/healthy) and their two- and three-way interactions as main effects using prediction error as the target variable. For each disease in the UK Biobank and AIBL datasets, we conducted statistical tests on samples comprising corresponding HC and diseased cohorts. For the AIBL study on dementia, we included both the MCI and AD cohorts in the same model.

**Figure 3:**
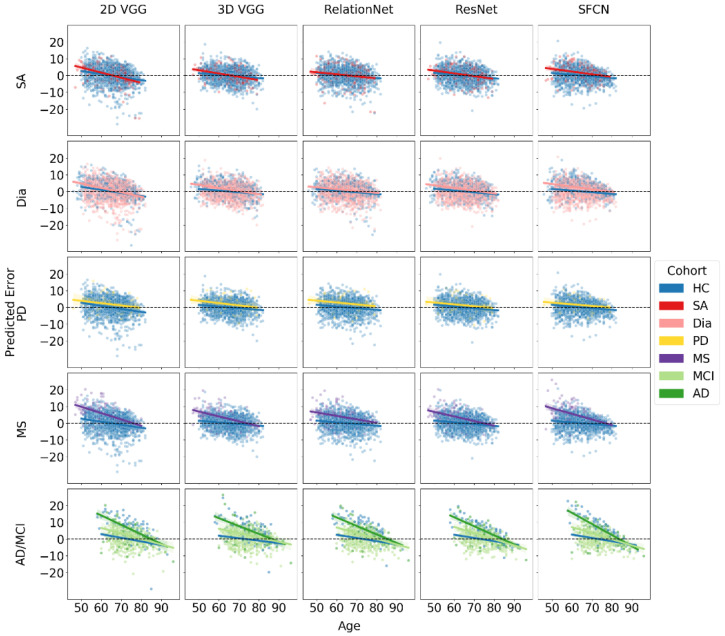
True age and prediction error for all five models for diseased subgroups and HC. The lines represent a fitted linear regression line for each subgroup, illustrating the relationship between age and prediction error for each model.

The results of the BAG estimation and age trendlines for each model and disease cohort are summarized in Table 6 and Figure 3. The table provides BAG values and age trend slope coefficient estimates along with their 95% CIs for each model architecture across different disease subsets.

For sleep apnea (SA), the ANOVA results showed that age, architecture, and their interaction were statistically significant (p<0.001), while the other effects were not (p>0.05). A pairwise comparison of marginal means for the cohort with SA revealed a statistically significant difference in estimated BAG between the SFCN architecture and all other architectures (p<0.001). The SFCN was the only model with a significant BAG of 1.59 years.

For subjects with diabetes, all models showed an increased BAG with respect to HC, which was marginally significant for the 2D VGG model, with a 95% CI close to zero, and the highest BAG (2.13 years) for the SFCN. Pairwise comparisons showed a statistically significant difference in the estimated BAG between the SFCN architecture and all other architectures (p<0.001). The ANOVA indicated that all three main effects and the two-way interaction between age and architecture were significant (p<0.001). Further, the interaction between cohort and architecture (p=0.010), cohort and age (p=0.032), and three-way interaction (p=0.020) were marginally significant.

For the MS subset, the LMEM showed that the age, architecture, their interaction, cohort, and the interaction between age and cohort were all statistically significant (p<0.001). The interaction between cohort and architecture was marginally significant (p=0.032). The BAG was statistically significantly different from zero for all models, ranging from over 4 years for the 2D VGG and SFCN models to about 2.7 years for the 3D VGG and ResNet models. Marginal means revealed significant differences between the two pairs (p<0.01).

For the Parkinson’s disease (PD) subset, age was the only statistically significant factor (p<0.001), with no significant marginal differences observed between models. Consequently, the differences in the BAG predictions among the models were not significant. The RelationNet model predicted the largest BAG at 2.3 years, while other models predicted BAGs ranging between 1.19 and 1.83 years. The estimated differences did not reach statistical significance for the ResNet (p=0.0560), were marginally significant for the SFCN (p=0.0361), and were significant for the other models (p<0.01).

For the combined cohort of MCI and AD from the AIBL dataset, ANOVA results indicated that age, cohort, architecture, the interactions between age and cohort, and age and architecture were all statistically significant (p<0.001). The BAG for MCI was consistent across all models, averaging around 2.3 years, with no significant differences observed between the models. In contrast, the estimated BAG for AD was approximately twice as large, ranging from 5.5 to 6.5 years.

Notably, all fitted regression lines exhibited negative slopes, indicating that for all significant BAG findings (where confidence intervals did not include zero), the absolute BAG decreased with increasing age. In datasets and models showing the largest BAGs, particularly for MS and AD, the diseased cohorts demonstrated a more pronounced negative trend in the slope coefficient compared to the HC subset. When comparing MCI and AD cohorts to the HC group, we observed a two-tiered trend in the regression slopes, suggesting that greater neurodegeneration is associated with steeper declines. This trend cannot be solely attributed to regression to the mean, indicating a genuine correlation between the BAG and chronological age.

## 4. Discussion

In this work, we examined the brain age gap (BAG) across various subject cohorts, i.e., healthy controls and seven diseased cohorts, and comparatively assessed the impact of five deep learning model architectures on BAG estimation. Evaluations were performed in accordance with the brain age standardized evaluation (BASE) [22]. Specifically, each model architecture’s weights were randomly initialized and trained five times, allowing for rigorous comparative statistical analyses using linear mixed-effects models and boosting prediction accuracy by averaging subsequent model predictions per subject. Our findings revealed several insights and potential limitations of these models, as well as the resulting implications of studying brain age in diseased populations.

### 4.1. Brain Age Gap across Subject Cohorts

As in many previous studies examining the BAG, we observed a regression to the mean effect [9,11,15,17]. Notably, the trend slope coefficient estimates were smaller in the multi-site dataset used for training compared to the new, previously unseen datasets and/or subject cohorts. Furthermore, in the new site or unseen site datasets, we observed a significant age offset, which was corrected by subtracting a constant value from all subsequent predictions, with the constant value being obtained as the mean error for the healthy controls subset (independently determined for UK Biobank and AIBL datasets). By setting the mean error of HC to zero, we could then directly compare the BAG between the diseased cohort subsets and the HCs across the datasets. Importantly, we did not regress out the dependence of the age prediction error on the chronological age, since such a correction could artificially inflate the correlation between brain age and chronological age, as well as random noise [11,45,46].

For datasets and models with the largest observed BAGs, such as those for MS and AD, the BAG was greater for younger subjects and decreased with age. This pattern has been observed in multiple related studies [4,11,47]. While this can be partially attributed to the regression to the mean effect, the pattern is clear and the estimated trend slope coefficients are substantially larger than those for HCs. Several factors could explain this phenomenon. For instance, survival bias may play a significant role, as older individuals with severe manifestations of diseases like MS and AD might be less likely to survive to advanced ages. Consequently, those who do survive may represent a subset with milder disease progression, leading to a smaller observed BAG in older age groups. Additionally, more aggressive treatment strategies (e.g., immunomodulatory therapy in MS) administered early in the disease course could influence the observed slope, as more disabled patients might benefit disproportionately from such interventions.

Supporting these explanations, Brier et al. [11] found that MS patients with a brain age lower than their chronological age were more likely to be female, less disabled, and have a higher age of symptom onset. Most notably, the presence of a high-efficacy disease-modifying therapy was associated with a brain age lower than chronological age.

A study by [48] supports the observed negative BAG slope by comparing the impact of normal aging versus MS-related atrophy across the brain and its substructures. The authors observed that during aging, the relative share of MS-specific brain volume change decreased, while the proportion of age-related brain volume change increased. Specifically, the rate of disease-attributed brain volume loss decreased from −0.38% per year at age 30 to −0.12% per year at age 60, while the rate of age-attributed brain volume change accelerated from −0.01% per year to −0.31% per year over the same age range. The deceleration of MS-specific brain volume change was more pronounced in the thalamus compared to the whole brain, where MS-specific atrophy decreased from −0.59% per year at age 30 to −0.05% per year at age 60.

### 4.2. Brain Age Gap across Different Model Architectures

Table 2 presents different estimations of the BAG for various models applied to the same subject cohort. Our results confirm the observation by [3] that the most accurate models, in terms of prediction error, do not necessarily provide the best separation between subjects in the HC and diseased cohorts. While the ResNet and 3D VGG models performed best on multi-site test datasets, the SFCN generally predicted a higher BAG. Among the models, the SFCN model was the only one to show a significantly different BAG from HCs across all diseased subgroups, including sleep apnea. Although other models also indicated a positive BAG sleep apnea, these results were not significantly different from zero. These observations indicate that the SFCN model demonstrates better separation between diseased cohorts and the HCs, making it more effective for distinguishing these groups. However, if the goal is to determine whether a specific disease or disorder actually results in an increased BAG, the findings for the sleep apnea cohort could be considered a potential false positive, as no other model found a significant BAG for this condition. Nonetheless, the particular result for the SFCN model is consistent with previous research, such as [20], which showed a positive correlation between BAG and indices quantifying an obstructive form of sleep apnea, and [49], which linked sleep-disordered breathing to a 26% higher likelihood of developing cognitive impairment.

It is crucial to recognize that non-significant results often remain unreported in scientific research, which can affect the interpretation and perceived robustness of findings. Our findings highlight significant differences in the absolute BAG values, depending on both the underlying disease and the neural network model architecture used. Even when all the models detected increased BAGs in the diseased cohorts compared to HCs, the exact magnitudes of these gaps varied considerably across models. Therefore, we should evaluate the magnitude of BAGs (and not the exact values) within each model, and possibly each dataset, to make relevant conclusions. For instance, patients with Alzheimer’s dementia exhibited the largest brain age gaps, reflecting the profound neurodegenerative impact of the disease. In contrast, those with MCI showed more subtle deviations, with the BAG for AD being up to three times higher than that for MCI.

### 4.3. Limitations

There are a few limitations in our study that should be noted. All subjects in the diseased dataset are middle-aged to older individuals, which may limit the generalizability of our findings to younger populations. Additionally, the study relies on cross-sectional data, restricting our ability to infer longitudinal changes in brain aging.

The varying sample sizes across disease cohorts might introduce biases, affecting the comparability of BAG results between different disease groups. While the models are comparable, the differences in sample sizes could impact the estimation of the BAG.

### 4.4. Future Recommendations

Based on the findings of this study, several key areas warrant further investigation to enhance the understanding and application of BAG as a biomarker for neurological diseases and disorders.

Firstly, it is essential to revalidate previous findings from the literature using well-calibrated models. Given that the magnitude of BAG varies across different deep learning architectures and pathologies, it is important to ensure that comparisons of BAG magnitudes are made within the same model predictions. This approach will help maintain consistency and accuracy in evaluating the relative impact of various conditions on brain aging.

Secondly, our study and others have consistently observed that the BAG decreases with increasing chronological age, particularly in conditions with large average BAG, such as multiple sclerosis, mild cognitive impairment, and Alzheimer’s dementia. This trend suggests that in older populations, the effects of aging may overshadow the influence of the underlying pathology on the BAG. This phenomenon raises important questions about the reliability of BAG as an early diagnostic biomarker, particularly in older age groups. Future research should focus on identifying the specific age intervals where BAG maintains a sufficient effect size to be clinically useful, and on understanding the underlying mechanisms that drive this age-related decline in BAG.

Finally, there is a need to explore the implications of these findings for clinical practice, particularly in the context of early diagnosis and monitoring of neurological conditions. Integrating advanced treatment data and conducting longitudinal studies will be crucial for refining these models, allowing for a more accurate assessment of brain aging and its association with various diseases. These efforts will ultimately contribute to the development of more effective diagnostic tools and therapeutic strategies, enhancing patient care and outcomes in neurological health.

## 5. Conclusions

In this study, we evaluated the brain age gap (BAG) across various neurological conditions using five deep learning models: 2D/3D VGG, RelationNet, ResNet, and SFCN. These models were trained on a multi-site dataset of healthy subjects and then applied to cohorts with sleep apnea, diabetes, multiple sclerosis, Parkinson’s disease, mild cognitive impairment, and Alzheimer’s disease.

Our results showed that the BAG varied across different architectures, with the SFCN model consistently producing the largest BAGs, indicating its superior sensitivity in distinguishing between healthy and diseased individuals. The error analysis revealed statistically significant positive BAGs across several conditions, with the largest discrepancies observed in Alzheimer’s disease (5.54 years with ResNet to 6.48 years with SFCN) and multiple sclerosis (2.67 years with 3D VGG to 4.24 years with 2D VGG). Notably, the BAG for Parkinson’s disease was significantly increased in all models except ResNet, and sleep apnea showed a significant increase only with the SFCN model.

Furthermore, we observed a trend where the BAG decreased with increasing chronological age, particularly in conditions like multiple sclerosis, mild cognitive impairment, and Alzheimer’s dementia, with trends ranging from −0.34 to −0.2 for multiple sclerosis, −0.37 to −0.26 for mild cognitive impairment, and −0.66 to −0.47 for Alzheimer’s dementia, compared to −0.18 to −0.1 in healthy controls. These results suggest that factors such as survival bias, disease progression, and model limitations play significant roles in brain age prediction.

In summary, the findings underscore the importance of selecting appropriate model architectures and considering disease-specific characteristics when interpreting BAG predictions. The integration of more advanced treatment data and longitudinal studies will be essential for refining these models and enhancing our understanding of brain aging across different neurological conditions.

## Figures and Tables

**Table 1:** Dataset statistics, such as span, mean age ($\mu_{\mathrm{age}}$), and standard deviation (${\mathrm{sd}}_{\mathrm{age}}$) in years, are provided per dataset for the included T1w subject scans in the train, test, and validation datasets.

Multi-Site Dataset	N	M%/F%	Age Interval	$\mu_{\mathrm{age}}\pm {\mathrm{sd}}_{\mathrm{age}}$
ABIDE I ^1^	161	88/12	18.0–48.0	25.7 ± 6.4
ADNI ^2,^*	248	51/49	60.0–90.0	76.2 ± 5.1
Cam-CAN [27,28] ^3^	624	49/51	18.0–88.0	54.2 ± 18.4
CC-359 [29] ^4^	349	49/51	29.0–80.0	53.5 ± 7.8
FCON 1000 ^5^	572	34/66	18.0–85.0	45.3 ± 18.9
IXI ^6^	472	47/53	20.1–86.2	49.0 ± 16.2
OASIS-2 [30] ^7^	78	28/72	60.0–95.0	75.6 ± 8.4
**Total**	2504	48/52	18.0–95.0	52.1 ± 19.1

Dataset information and download at ^1^ ABIDE I: http://fcon_1000.projects.nitrc.org/indi/abide/abide_I.html, ^2^ ADNI: http://adni.loni.usc.edu/, ^3^ Cam-CAN: https://camcan-archive.mrc-cbu.cam.ac.uk/dataaccess/, ^4^ CC-359: https://sites.google.com/view/calgary-campinas-dataset/download, ^5^ FCON 1000: http://fcon_1000.projects.nitrc.org/indi/enhanced/neurodata.html, ^6^ IXI: https://brain-development.org/ixi-dataset/, ^7^ OASIS-2: https://www.oasis-brains.org/. All links accessed on 13 July 2024. * Data used in the preparation of this article were obtained from the Alzheimer’s Disease Neuroimaging Initiative (ADNI) database (adni.loni.usc.edu). The ADNI was launched in 2003 as a public–private partnership, led by Principal Investigator Michael W. Weiner, MD. The primary goal of ADNI has been to test whether serial magnetic resonance imaging (MRI), positron emission tomography (PET), other biological markers, and clinical and neuropsychological assessment can be combined to measure the progression of mild cognitive impairment (MCI) and early Alzheimer’s disease (AD).

**Table 2:** Subject count, sex, and age statistics (in years) by subset of the UK Biobank and the AIBL datasets. Abbreviations: healthy controls (HC), sleep apnea (SA), diabetes (Dia), multiple sclerosis (MS), Parkinson’s disease (PD), mild cognitive impairment (MCI), Alzheimer’s dementia (AD).

	UK Biobank ^1^					AIBL ^2^		
	HC	SA	Dia	MS	PD	HC	MCI	AD
**N**	1347	201	454	114	58	440	95	95
**sex** M %	45	81	65	26	36	43	54	44
**age** μ±σ	64.9±7.1	65.2±7.4	64.8±8.6	60.5±7.2	64.6±7.7	73.5±6.2	75.5±7.4	75.1±7.6
**age interval** [min,max]	[50.0, 82.0]	[47.0, 79.0]	[46.0, 81.0]	[47.0, 80.0]	[46.0,79.0]	[60.0, 92.0]	[60.0, 96.0]	[58.0, 93.0]

Dataset information and download at: ^1^ UK Biobank: https://www.ukbiobank.ac.uk/, ^2^ AIBL: https://adni.loni.usc.edu/category/aibl-study-data/. All links accessed on 13 July 2024.

**Table 3:** The final hyperparameter values for each model architecture. Hyperparameters were selected based on the proposed values in the original papers. Hyperparameters marked with * were reevaluated.

	2D VGG	3D VGG	RelationNet	ResNet	SFCN
**Input Size**	157×189×15	157×189×170	157×189×170	157×189×170	157×189×170
* Batch Size	32	16	16	16	8
**Loss Function**	L1	L1	L1	L1	KLD
* Learning Rate (LR)	$1\times 10^{-3}$	$1\times 10^{-4}$	$1\times 10^{-4}$	$1\times 10^{-3}$	$1\times 10^{-2}$
**LR Decay**	$1\times 10^{-4}$	3%	50%	50%	70%
**LR Decay Step**	1	1	35	30	30
**Weight Decay**	$10^{-3}$	0	0	0	$10^{-3}$
**Momentum**	0.9	0.9	0.9	0.9	0.9
**Epochs**	400	110	160	130	110

**Table 4:** Evaluation of five model architectures on the multi-site test set of healthy subjects. The best metric result with respect to model architecture (in *rows*) is marked in **bold**. All numbers are in years.

	Accuracy		Robustness	LMEM
	ME (SD)	MAE (SD)	mMAE	Age Trend Slope Coefficient [CI]
**2D VGG**	−0.80±4.81	3.72±3.14	4.36	−0.102(−0.128,−0.076)
**3D VGG**	−0.03±3.86	2.96±2.47	3.63	−0.036(−0.062,−0.010)
**RelationNet**	−0.22±4.14	3.06±2.79	3.92	−0.053(−0.079,−0.027)
**ResNet**	−0.41±3.84	2.91±2.53	3.78	−0.050(−0.076,−0.024)
**SFCN**	−0.46±4.21	3.25±2.70	4.40	−0.082(−0.108,−0.056)

**Table 5:** Accuracy and robustness results on the UK Biobank dataset. The first three columns represent the results without offset correction, and the second three columns with offset correction. The best metric result with respect to model architecture (in *rows*) is marked in **bold**. All numbers are in years.

	Without Offset Corr.			With Offset Corr.			
	Accuracy		Robustness	Accuracy		Robustness	LMEM
	ME (SD)	MAE (SD)	mMAE	ME (SD)	MAE (SD)	mMAE	Age Trend Estimate [CI]
**2D VGG**	−1.64±5.65	4.38±3.93	6.38	0.0±5.65	4.31±3.65	5.27	−0.181[−0.216,−0.147]
**3D VGG**	−1.96±4.26	3.68±2.90	5.82	0.0±4.26	3.35±2.62	4.32	−0.099[−0.134,−0.065]
**RelationNet**	−2.55±4.78	4.19±3.43	6.13	0.0±4.78	3.73±2.98	4.22	−0.101[−0.136,−0.067]
**ResNet**	−0.71±4.45	3.56±2.76	**4.38**	0.0±4.45	3.55±2.69	**3.94**	−0.109[−0.143,−0.074]
**SFCN**	−1.16±4.30	3.42±2.86	5.39	0.0±4.30	3.32±2.74	4.38	−0.098[−0.132,−0.063]

**Table 6:** Brain age gap (BAG) values and age trend slope coefficient estimates, along with their 95% confidence intervals (CI) are provided for each model architecture across different disease cohorts, including sleep apnea (SA), diabetes (Dia), multiple sclerosis (MS), and Parkinson’s disease (PD) from the UK Biobank, and mild cognitive impairment (MCI) and Alzheimer’s disease (AD) from the AIBL dataset. The BAG values indicate the difference in predicted brain age between diseased groups and healthy controls, while the age trend estimates reflect how the prediction errors change with respect to the actual age. The significant BAGs, where the corresponding CI does not contain zero, are marked in **bold**.

Cohort	Metric	2D VGG	3D VGG	RelationNet	ResNet	SFCN
**SA**	BAG	0.25(−0.44,0.93)	0.25(−0.44,0.93)	0.16(−0.52,0.85)	0.48(−0.20,1.17)	1.59(0.91,2.28)
	Age trend	−0.30(−0.39,−0.22)	−0.20(−0.28,−0.11)	−0.12(−0.21,−0.03)	−0.17(−0.25,−0.08)	−0.16(−0.25,−0.07)
**Dia**	BAG	0.81(0.030,1.31)	1.12(0.62,1.63)	0.79(0.29,1.29)	1.26(0.76,1.77)	2.13(1.62,2.63)
	Age trend	−0.26(−0.31,−0.21)	−0.19(−0.24,−0.13)	−0.11(−0.16,−0.06)	−0.16(−0.21,−0.11)	−0.16(−0.21,−0.11)
**MS**	BAG	4.24(3.22,5.25)	2.67(1.65,3.68)	3.38(2.37,4.40)	2.73(1.71,3.75)	4.05(3.04,5.06)
	Age trend	−0.38(−0.50,−0.26)	−0.29(−0.41,−0.17)	−0.20(−0.32,−0.09)	−0.28(−0.40,−0.16)	−0.34(−0.46,−0.22)
**PD**	BAG	1.80(0.58,3.02)	1.83(0.61,3.05)	2.31(1.09,3.53)	1.19(−0.03,2.41)	1.30(0.08,2.52)
	Age trend	−0.14(−0.30,0.02)	−0.14(−0.30,0.02)	−0.11(−0.27,0.04)	−0.11(−0.27,0.04)	−0.10(−0.26,0.06)
**MCI**	BAG	2.13(0.95,3.31)	2.59(1.42,3.77)	2.31(1.13,3.49)	2.19(1.01,3.37)	2.16(0.98,3.33)
	Age trend	−0.33(−0.45,−0.21)	−0.26(−0.38,−0.14)	−0.34(−0.46,−0.22)	−0.37(−0.49,−0.25)	−0.36(−0.48,−0.24)
**AD**	BAG	6.30(5.13,7.47)	5.96(4.79,7.13)	5.92(4.75,7.09)	5.54(4.37,6.71)	6.48(5.31,7.65)
	Age trend	−0.57(−0.68,−0.45)	−0.47(−0.59,−0.35)	−0.51(−0.62,−0.39)	−0.54(−0.66,−0.42)	−0.66(−0.78,−0.55)

## Data Availability

Public datasets can be acquired from the dataset providers. For more details, please see Table 4.

## Abbreviations

The following abbreviations are used in this manuscript:

T1w	T1-weighted
MR	Magnetic Resonance
BAG	Brain Age Gap
HC	Healthy Controls
SA	Sleep Apnea
Dia	Diabetes
MS	Multiple Sclerosis
PD	Parkinson’s Disease
MCI	Mild Cognitive Impairment
AD	Alzheimer’s Dementia
MNI	Montreal Neurological Institute
KLD	Kullback–Leibler Divergence
SGD	Stochastic Gradient Descent
BASE	Brain Age Standardized Evaluation
MAE	Mean Absolute Error
LMEMs	Linear Mixed-Effects Models
ANOVA	Analysis of Variance
EMM	Estimated Marginal Means

## References

[1] Sajedi H., Pardakhti N. (2019). Age Prediction Based on Brain MRI Image: A Survey. J. Med. Syst..

[2] Cole J.H., Franke K., Cherbuin N., Moskalev A. (2019). Quantification of the Biological Age of the Brain Using Neuroimaging. Biomarkers of Human Aging.

[3] Bashyam V.M., Erus G., Doshi J., Habes M., Nasrallah I.M., Truelove-Hill M., Srinivasan D., Mamourian L., Pomponio R., Fan Y. (2020). MRI signatures of brain age and disease over the lifespan based on a deep brain network and 14468 individuals worldwide. Brain.

[4] Beheshti I., Mishra S., Sone D., Khanna P., Matsuda H. (2020). T1-weighted MRI-driven Brain Age Estimation in Alzheimer’s Disease and Parkinson’s Disease. Aging Dis..

[5] Charissé D., Erus G., Pomponio R., Gorges M., Schmidt N., Schneider C., Liepelt-Scarfone I., Riedel O., Reetz K., Schulz J.B. (2022). Brain age and Alzheimer’s-like atrophy are domain-specific predictors of cognitive impairment in Parkinson’s disease. Neurobiol. Aging.

[6] Chen C.-L., Cheng S.-Y., Montaser-Kouhsari L., Wu W.-C., Hsu Y.-C., Tai C.-H., Tseng W.-Y.I., Kuo M.-C., Wu R.-M. (2024). Advanced brain aging in Parkinson’s disease with cognitive impairment. NPJ Park. Dis..

[7] Franke K., Gaser C. (2012). Longitudinal changes in individual BrainAGE in healthy aging, mild cognitive impairment, and Alzheimer’s disease. GeroPsych J. Gerontopsychol. Geriatr. Psychiatry.

[8] Lee J., Burkett B.J., Min H.K., Senjem M.L., Lundt E.S., Botha H., Graff-Radford J., Barnard L.R., Gunter J.L., Schwarz C.G. (2022). Deep learning-based brain age prediction in normal aging and dementia. Nat. Aging.

[9] Cheng J., Liu Z., Guan H., Wu Z., Zhu H., Jiang J., Wen W., Tao D., Liu T. (2021). Brain Age Estimation From MRI Using Cascade Networks With Ranking Loss. IEEE Trans. Med. Imaging.

[10] Høgestøl E.A., Kaufmann T., Nygaard G.O., Beyer M.K., Sowa P., Nordvik J.E., Kolskår K., Richard G., Andreassen O.A., Harbo H.F. (2019). Cross-Sectional and Longitudinal MRI Brain Scans Reveal Accelerated Brain Aging in Multiple Sclerosis. Front. Neurol..

[11] Brier M.R., Li Z., Ly M., Karim H.T., Liang L., Du W., McCarthy J.E., Cross A.H., Benzinger T.L.S., Naismith R.T. (2023). “Brain age” predicts disability accumulation in multiple sclerosis. Ann. Clin. Transl. Neurol..

[12] Cole J.H., Raffel J., Friede T., Eshaghi A., Brownlee W.J., Chard D., Stefano N.D., Enzinger C., Pirpamer L., Filippi M. (2020). Longitudinal Assessment of Multiple Sclerosis with the Brain-Age Paradigm. Ann. Neurol..

[13] Franke K., Gaser C., Manor B., Novak V. (2013). Advanced BrainAGE in older adults with type 2 diabetes mellitus. Front. Aging Neurosci..

[14] Sun H., Paixao L., Oliva J.T., Goparaju B., Carvalho D.Z., van Leeuwen K.G., Akeju O., Thomas R.J., Cash S.S., Bianchi M.T. (2019). Brain age from the electroencephalogram of sleep. Neurobiol. Aging.

[15] Kolbeinsson A., Filippi S., Panagakis Y., Matthews P.M., Elliott P., Dehghan A., Tzoulaki I. (2020). Accelerated MRI-predicted brain ageing and its associations with cardiometabolic and brain disorders. Sci. Rep..

[16] Cole J.H. (2020). Multimodality neuroimaging brain-age in UK biobank: Relationship to biomedical, lifestyle, and cognitive factors. Neurobiol. Aging.

[17] Jha M.K., Chin Fatt C.R., Minhajuddin A., Mayes T.L., Berry J.D., Trivedi M.H. (2022). Accelerated brain aging in individuals with diabetes: Association with poor glycemic control and increased all-cause mortality. Psychoneuroendocrinology.

[18] Zhang T., Shaw M., Cherbuin N. (2022). Association between Type 2 Diabetes Mellitus and Brain Atrophy: A Meta-Analysis. Diabetes Metab. J..

[19] Wang D.Q., Wang L., Wei M.M., Xia X.S., Tian X.L., Cui X.H., Li X. (2020). Relationship Between Type 2 Diabetes and White Matter Hyperintensity: A Systematic Review. Front. Endocrinol..

[20] Weihs A., Frenzel S., Wittfeld K., Obst A., Stubbe B., Habes M., Szentkirályi A., Berger K., Fietze I., Penzel T. (2021). Associations between sleep apnea and advanced brain aging in a large-scale population study. Sleep.

[21] Bittner N., Jockwitz C., Franke K., Gaser C., Moebus S., Bayen U.J., Amunts K., Caspers S. (2021). When your brain looks older than expected: Combined lifestyle risk and BrainAGE. Brain Struct. Funct..

[22] Dular L., Špiclin Ž. (2024). BASE: Brain Age Standardized Evaluation. NeuroImage.

[23] Baecker L., Dafflon J., da Costa P.F., Garcia-Dias R., Vieira S., Scarpazza C., Calhoun V.D., Sato J.R., Mechelli A., Pinaya W.H.L. (2021). Brain age prediction: A comparison between machine learning models using region- and voxel-based morphometric data. Hum. Brain Mapp..

[24] Beheshti I., Ganaie M.A., Paliwal V., Rastogi A., Razzak I., Tanveer M. (2022). Predicting Brain Age Using Machine Learning Algorithms: A Comprehensive Evaluation. IEEE J. Biomed. Health Infor..

[25] Kaufmann T., van der Meer D., Doan N.T., Schwarz E., Lund M.J., Agartz I., Alnæs D., Barch D.M., Baur-Streubel R., Bertolino A. (2019). Common brain disorders are associated with heritable patterns of apparent aging of the brain. Nat. Neurosci..

[26] More S., Antonopoulos G., Hoffstaedter F., Caspers J., Eickhoff S.B., Patil K.R. (2023). Brain-age prediction: A systematic comparison of machine learning workflows. NeuroImage.

[27] Shafto M.A., Tyler L.K., Dixon M., Taylor J.R., Rowe J.B., Cusack R., Calder A.J., Marslen-Wilson W.D., Duncan J., Dalgleish T. (2014). The Cambridge Centre for Ageing and Neuroscience (Cam-CAN) study protocol: A cross-sectional, lifespan, multidisciplinary examination of healthy cognitive ageing. BMC Neurol..

[28] Taylor J.R., Williams N., Cusack R., Auer T., Shafto M.A., Dixon M., Tyler L.K., Henson R.N., Cam-CAN (2017). The Cambridge Centre for Ageing and Neuroscience (Cam-CAN) data repository: Structural and functional MRI, MEG, and cognitive data from a cross-sectional adult lifespan sample. NeuroImage.

[29] Souza R., Lucena O., Garrafa J., Gobbi D., Saluzzi M., Appenzeller S., Rittner L., Frayne R., Lotufo R. (2018). An open, multi-vendor, multi-field-strength brain MR dataset and analysis of publicly available skull stripping methods agreement. NeuroImage.

[30] Marcus D.S., Fotenos A.F., Csernansky J.G., Morris J.C., Buckner R.L. (2010). Open Access Series of Imaging Studies: Longitudinal MRI Data in Nondemented and Demented Older Adults. J. Cogn. Neurosci..

[31] Miller K.L., Alfaro-Almagro F., Bangerter N.K., Thomas D.L., Yacoub E., Xu J., Bartsch A.J., Jbabdi S., Sotiropoulos S.N., Andersson J.L.R. (2016). Multimodal population brain imaging in the UK Biobank prospective epidemiological study. Nat. Neurosci..

[32] Pucak M.L., Carroll K.A.L., Kerr D.A., Kaplin A.L. (2007). Neuropsychiatric manifestations of depression in multiple sclerosis: Neuroinflammatory, neuroendocrine, and neurotrophic mechanisms in the pathogenesis of immune-mediated depression. Dialogues Clin. Neurosci..

[33] Chikatimalla R., Dasaradhan T., Koneti J., Cherukuri S., Kalluru R., Gadde S. (2022). Depression in Parkinson’s Disease: A Narrative Review. Cureus.

[34] Ellis K.A., Bush A.I., Darby D., De Fazio D., Foster J., Hudson P., Lautenschlager N.T., Lenzo N., Martins R.N., Maruff P. (2009). The Australian Imaging, Biomarkers and Lifestyle (AIBL) study of aging: Methodology and baseline characteristics of 1112 individuals recruited for a longitudinal study of Alzheimer’s disease. Int. Psychogeriatr..

[35] Dular L., Pernuš F., Špiclin Ž. (2024). Extensive T1-weighted MRI preprocessing improves generalizability of deep brain age prediction models. Comput. Biol. Med..

[36] Manjón J.V., Coupé P., Martí-Bonmatí L., Collins D.L., Robles M. (2010). Adaptive non-local means denoising of MR images with spatially varying noise levels. J. Magn. Reson. Imaging.

[37] Modat M., Cash D.M., Daga P., Winston G.P., Duncan J.S., Ourselin S. (2014). Global image registration using a symmetric block-matching approach. J. Med. Imaging.

[38] Tustison N.J., Avants B.B., Cook P.A., Zheng Y., Egan A., Yushkevich P.A., Gee J.C. (2010). N4ITK: Improved N3 bias correction. IEEE Trans. Med. Imaging.

[39] Huang T., Chen H., Fujimoto R., Ito K., Wu K., Sato K., Taki Y., Fukuda H., Aoki T. Age estimation from brain MRI images using deep learning. Proceedings of the 2017 IEEE 14th International Symposium on Biomedical Imaging (ISBI 2017).

[40] Cole J.H., Poudel R.P.K., Tsagkrasoulis D., Caan M.W.A., Steves C., Spector T.D., Montana G. (2017). Predicting brain age with deep learning from raw imaging data results in a reliable and heritable biomarker. NeuroImage.

[41] He S., Feng Y., Grant P.E., Ou Y. (2022). Deep Relation Learning for Regression and Its Application to Brain Age Estimation. IEEE Trans. Med. Imaging.

[42] Hu L., Wan Q., Huang L., Tang J., Huang S., Chen X., Bai X., Kong L., Deng J., Liang H. (2023). MRI-based brain age prediction model for children under 3 years old using deep residual network. Brain Struct. Funct..

[43] Dartora C., Marseglia A., Mårtensson G., Rukh G., Dang J., Muehlboeck J.S., Wahlund L.O., Moreno R., Barroso J., Ferreira D. (2024). A deep learning model for brain age prediction using minimally preprocessed T1w images as input. Front. Aging Neurosci..

[44] Peng H., Gong W., Beckmann C.F., Vedaldi A., Smith S.M. (2021). Accurate brain age prediction with lightweight deep neural networks. Med. Image Anal..

[45] Butler E.R., Chen A., Ramadan R., Le T.T., Ruparel K., Moore T.M., Satterthwaite T.D., Zhang F., Shou H., Gur R.C. (2021). Pitfalls in brain age analyses. Hum. Brain Mapp..

[46] de Lange A.M.G., Anatürk M., Rokicki J., Han L.K.M., Franke K., Alnæs D., Ebmeier K.P., Draganski B., Kaufmann T., Westlye L.T. (2022). Mind the gap: Performance metric evaluation in brain-age prediction. Hum. Brain Mapp..

[47] Karim H.T., Aizenstein H.J., Mizuno A., Ly M., Andreescu C., Wu M., Hong C.H., Roh H.W., Park B., Lee H. (2022). Independent replication of advanced brain age in mild cognitive impairment and dementia: Detection of future cognitive dysfunction. Mol. Psychiatry.

[48] Azevedo C.J., Cen S.Y., Jaberzadeh A., Zheng L., Hauser S.L., Pelletier D. (2019). Contribution of normal aging to brain atrophy in MS. Neurol. Neuroimmunol. Neuroinflamm..

[49] Leng Y., McEvoy C.T., Allen I.E., Yaffe K. (2017). Association of Sleep-Disordered Breathing with Cognitive Function and Risk of Cognitive Impairment: A Systematic Review and Meta-analysis. JAMA Neurol..

